# Evidence for Impact: Evaluating Core Treatment Goals in Pediatric Palliative Inpatient Care

**DOI:** 10.3390/children12121606

**Published:** 2025-11-26

**Authors:** Pia Schmidt, Julia Wager, Gina Warrender, Dörte Garske, Andrea Beissenhirtz, Boris Zernikow, Larissa Kubek

**Affiliations:** 1 Pediatric Palliative Care Centre, Children’s and Adolescents’ Hospital Datteln, Dr.-Friedrich-Steiner-Str. 5, 45711 Datteln, Germany; j.wager@deutsches-kinderschmerzzentrum.de (J.W.); g.warrender@kinderklinik-datteln.de (G.W.); d.garske@kinderklinik-datteln.de (D.G.); a.beissenhirtz@kinderklinik-datteln.de (A.B.); b.zernikow@kinderklinik-datteln.de (B.Z.); 2 Department of Children’s Pain Therapy and Paediatric Palliative Care, Faculty of Health, School of Medicine, Witten/Herdecke University, Alfred-Herrhausen-Straße 50, 58455 Witten, Germany; 3 PedScience Research Institute, Herdieckstr. 5b, 45711 Datteln, Germany; l.kubek@pedscience.de

**Keywords:** pediatric palliative care, outcome measures, quality of care, treatment goals, inpatient

## Abstract

**Background**: In light of the increasing importance of measuring, assuring, and improving the quality of care, this study aimed to evaluate the outcome of care provided at a specialized inpatient PPC unit in consideration of the three PPC core treatment goals: (i) reduction in symptom burden, (ii) improvement of the quality of life, and (iii) strengthening caregiver competency. **Methods**: Prospective single-center study. Data were collected using the QUASI, a shorter version of the validated FAmily-CEnTered multidimenSional Outcome measure For Pediatric Palliative Care (FACETS-OF-PPC). From 20 October 2020 to 31 December 2024, QUASI was completed by nurses at admission (pre-survey) to an 8-bed specialized inpatient PPC unit and again on the day of discharge (post-survey). Descriptive statistics and the Wilcoxon signed-rank test were used to identify differences between pre- and post-surveys. In addition to outcome evaluation, psychometric properties of the Caregiver Competencies subscale were assessed (internal consistency and confirmatory factor analysis). **Results**: Pre- and post-data of *n* = 574 patients have been assessed. Among patients with initially high symptom load, significant improvement in all assessed symptoms could be achieved. Patients with initially low symptom load showed significant improvements in agitation (*p* = 0.043), pain (*p* < 0.001), sleep disturbances (*p* < 0.001), and other symptoms (*p* = 0.015). Mean scores for the items regarding quality of life also improved significantly between admission and discharge. Caregiver’s competency also increased significantly (*p* < 0.001). **Conclusions**: Findings suggest that the QUASI can serve as a feasible approach for documenting PPC quality, as it covers core dimensions of PPC very effectively, with the potential to inform clinical practice. Further validation is warranted.

## 1. Introduction

Pediatric palliative care (PPC) addresses the complex and evolving needs of children with life-limiting conditions and their families, aiming to improve their quality of life throughout the course of illness. In addition to symptom control, PPC provides social, emotional, and spiritual support, aiming to enhance the quality of life for the entire care unit, i.e., the patient and their family. It therefore adopts a holistic care approach, addressing and alleviating physical, psychological, social, and spiritual suffering [1,2,3].

In general, PPC patients suffer from rare or even undiagnosed neurological, genetic/congenital, neuromuscular, metabolic, or oncological diseases. Due to their age or developmental stage, they are often unable to communicate verbally, and their symptoms are extremely heterogeneous. These symptoms include respiratory problems, agitation, pain, seizures, and sleep disturbances [4]. As the complexity of care in PPC patients does not inevitably correspond to the degree of medical complexity [5], the multidimensional needs of PPC patients are strongly expressed through nursing diagnoses, which capture aspects such as symptom management, caregiver support, and quality of life. Thus, many PPC patients require complex medical technologies, such as home ventilation, resulting in a high level of nursing complexity that needs to be addressed.

PPC is addressed to children, adolescents, and young adults, with most living at home with their families [6]. They receive support from various care providers, each of which has a different approach to supporting PPC patients and their families. In addition to general PPC, which is provided by professionals such as pediatricians, pediatric nursing services, or short-term care facilities, there are specialized PPC services, including children and adolescents’ hospices, specialized pediatric palliative home care (SPPHC) teams, and PPC units in Germany. PPC units are considered if PPC patients can no longer be cared for at home, such as due to crises or high symptom burden. The first specialized inpatient PPC unit in Germany was established in 2010 at one of the largest university-affiliated pediatric hospitals. It provides highly specialized PPC for annually approximately *N* = 170 patients with mainly neurological underlying diseases and their families Up to eight patients can be accommodated in single rooms or apartments. Healthcare providers from a variety of professional backgrounds collaborate in accordance with the bio-psycho-social-spiritual treatment approach to ensure high-quality care. Therefore, in addition to the child’s current state of health, the general well-being of the entire family is always considered to achieve the core PPC treatment goals [2].

In light of the increasing importance of measuring, assuring, and improving the quality of care, this study aimed to evaluate the outcome of care provided at a specialized inpatient PPC unit in consideration of the three PPC core treatment goals: (i) reduction in symptom burden, (ii) improvement of the quality of life of both the child and their family, and (iii) strengthening caregiver competency. To this end, data were collected using the QUASI, a shorter version of the validated FAmily-CEnTered multidimenSional Outcome measure For Pediatric Palliative Care (FACETS-OF-PPC) [7].

## 2. Materials and Methods

### 2.1. Design

A prospective single-center study was conducted to assess the outcome of care provided at an 8-bed specialized inpatient PPC unit. Data were collected systematically from 20 October 2020 to 31 December 2024 as part of a structured quality assurance process.

### 2.2. Participants

All patients admitted to the PPC unit of the Children’s and Adolescents’ Hospital Datteln, Witten/Herdecke University, were eligible for participation. In line with the unit’s scope, which serves children, adolescents, and selected young adults with ongoing pediatric palliative needs, the study included all admissions to the unit during the study period so that no prespecified age limits were applied. Exclusion criteria included (a) inpatient stay of less than three days, (b) internal or external transfers before completion of three days, and (c) death during the inpatient stay.

Data collection using the QUASI was part of the unit’s routine quality assurance activities, in accordance with institutional procedures and applicable legal regulations. Families were informed upon admission that anonymized documentation of care processes and outcomes may be used for internal evaluation and research purposes. No additional data were collected beyond standard clinical documentation, and no identifiable patient or family data were used for analysis. Families were given the opportunity to opt out of the use of their anonymized data at any time; however, no family made use of this option during the study period. This approach was approved by the responsible institutional review board (Ethics Committee of the Children’s and Adolescents’ Hospital Datteln, Witten/Herdecke University, approval code: 2025/07/16/PS).

### 2.3. QUASI

To assess quality of care from a multidimensional perspective, the quality measurement tool QUASI was applied. The QUASI questionnaire comprises 22 items, 18 of which are drawn directly from the validated FAmily-CEnTered multidimenSional Outcome measure For Pediatric Palliative Care (FACETS-OF-PPC) [7], and an additional four items to assess the global dimension of care.

The FACETS-OF-PPC was developed specifically for pediatric patients with life-threatening or life-limiting conditions. It is available in both parent and healthcare provider versions and has been validated in multiple prospective multicenter studies and adheres to the EAPC White Paper recommendations regarding the key parameters of outcome measurement in PC [8].

The selection of the 18 items aimed to cover key constructs relevant to PPC while ensuring feasibility for integration into daily nursing documentation. Four additional items were included to assess global dimensions of care: (1) the child’s overall symptom burden, (2) the child’s general health status, (3) the quality of life of the child, and (4) the quality of life of the entire family.

In this study, the psychometric properties of the “Caregiver Competencies” subscale were examined. Internal consistency was assessed using Cronbach’s alpha and demonstrated acceptable internal consistency (Cronbach’s α = 0.79). A confirmatory factor analysis (CFA) further supported the expected one-factor structure. The model showed excellent fit, χ^2^(3) = 638.20, *p* < 0.001; CFI = 1.00; TLI = 1.00; RMSEA = 0.00; SRMR = 0.000. According to commonly accepted cut-offs (CFI/TLI ≥ 0.95; RMSEA ≤ 0.06; SRMR ≤ 0.08; [9]), these values indicate an excellent fit between the hypothesized model and the observed data. The three items loaded strongly on the latent factor, with standardized factor loadings of 0.64 (family able to independently alleviate symptoms), 0.92 (family prepared for crisis), and 0.70 (family has a clear idea for medical emergency). All loadings exceeded the recommended threshold of 0.40, suggesting a coherent and meaningful subscale structure. These findings provide further psychometric support for this subscale within the QUASI instrument.

The QUASI was completed by nurses as part of routine care to enable consistent documentation and to minimize burden on families; the results therefore reflect nurse-reported assessments.

The items used were grouped into three thematic sections: Part A focuses on the family’s current situation and includes the “Caregiver Competencies” subscale, consisting of the following three items: “If necessary, the family is able to independently take measures to alleviate their child’s symptoms”, “The family is prepared for the child’s crisis”, and “The family has a clear idea of what should be done for their child in a medical emergency”. While the patient’s symptoms are focused in Part B, Part C provides a global assessment of the child’s and family’s general condition.

Items in Parts A and B are rated using a 6-point Likert scale (1 = completely disagree/not present; 5 = completely agree/very strongly pronounced). Items in Part C are rated on a 10-point scale (1 = very bad/not present; 10 = very strongly pronounced/very good).

To characterize the patient population, routinely collected clinical data were extracted, including gender, date of admission and discharge, and the underlying disease of the child according to Feudtner’s system of pediatric complex chronic conditions (version 3) [10].

### 2.4. Data Collection

Data were collected over a four-year period (20 October 2020–31 December 2024). QUASI was completed by nurses on the third day after patient admission (pre-survey) and again on the day of discharge (post-survey). Although some children were admitted more than once during the study period, each admission was treated as a separate case and assessed independently. For the sake of readability and clarity, all cases are referred to as ‘patients’ throughout the manuscript. Whenever possible, the pre- and post-survey were conducted by the same nurse. The patient’s routine data were extracted from the patient’s file.

### 2.5. Analyses

Only data from patients for whom both pre- and post-values were available were included in the analysis.

All analyses were performed using IBM SPSS Statistics, version 29. Patient characteristics were analyzed descriptively. Items 3, 4, and 5 of the QUASI Part A, which constitute the Scale Caregiver Competencies, were tested for internal consistency using Cronbach’s alpha. Based on baseline symptom severity, patients were stratified into two groups: low symptom load (very weakly pronounced and weakly pronounced) and high symptom load (moderately pronounced, strongly pronounced, and very strongly pronounced). Normality of distribution was assessed using the Shapiro–Wilk test and visual inspection of histograms. In case of non-normal distribution, we used the non-parametric Wilcoxon signed-rank test for inferential statistics. To enhance interpretability, we present both mean (±SD) and median where applicable.

## 3. Results

The QUASI was used for all patients admitted to the PPC unit during the study period (*N* = 682). Complete pre- and post-assessment data were available for *n* = 574 (84.2%) patients. No data were available for *n* = 108 (15.8%) patients based on the defined exclusion criteria (inpatient stay of less than three days, *n* = 60; internal or external transfers before completion of three days, *n* = 1; death during the inpatient stay, *n* = 9) or because due to a high workload nurses did not complete the QUASI (*n* = 38).

The patients’ mean age was 8.9 years (range 0.1–31.0 years, SD: 6.3); *n* = 244 (42.5%) were female and *n* = 330 (57.5%) were male. The mean duration of inpatient stay was 16.1 days (range 2–201 days, SD: 16.5). The patients’ underlying diseases are presented in Table 1.

Table 2 provides an overview of the average pre- and post-values of all QUASI items.

**Figure 1:**
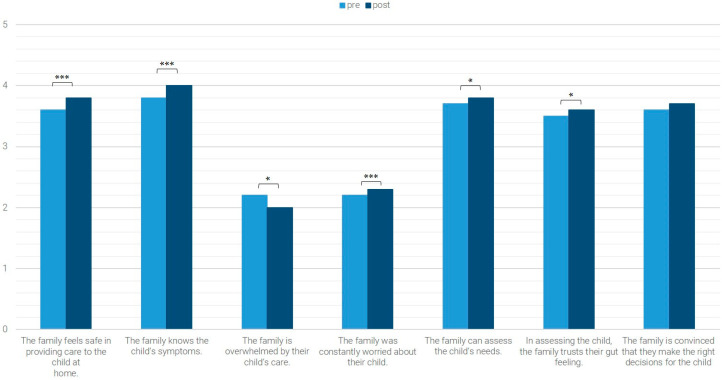
Current situation of the family * *p* < 0.05; *** *p* < 0.001.

**Figure 2:**
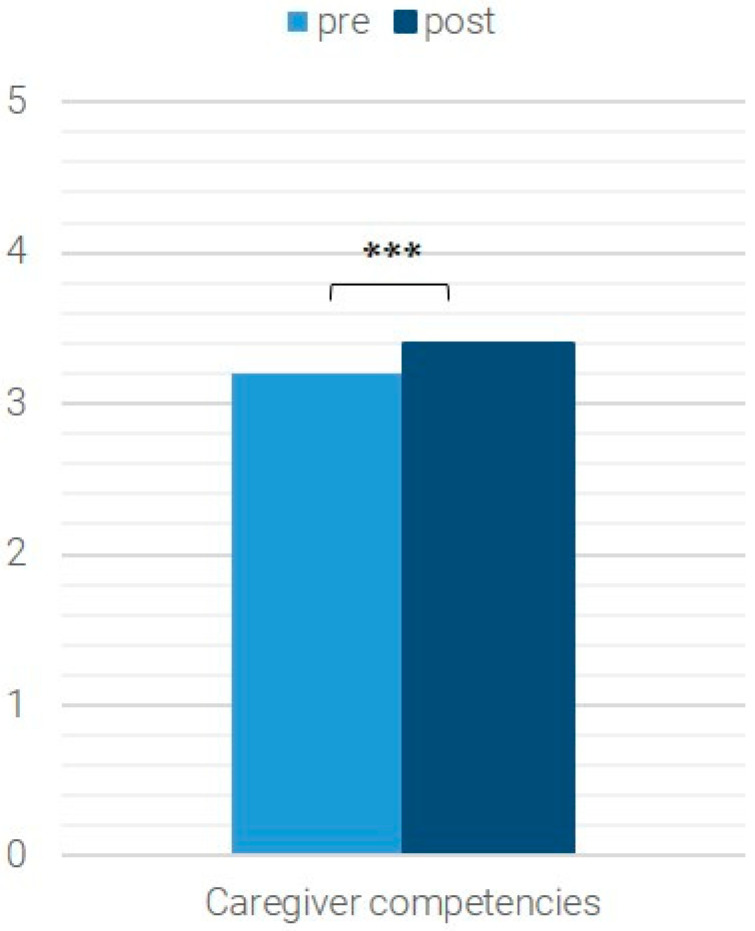
Caregiver competencies; *** *p* < 0.001.

**Figure 3:**
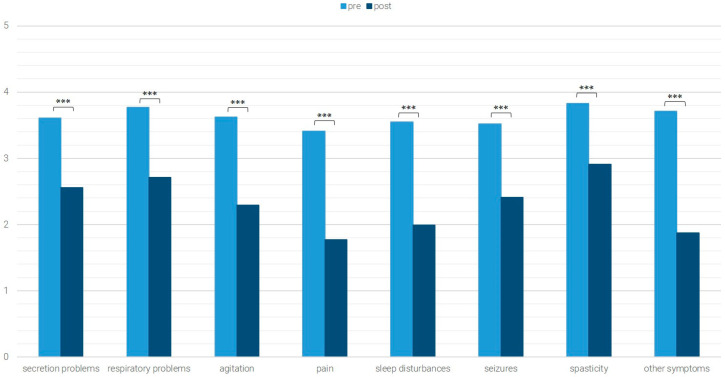
Occurrence of symptoms with an initially at least moderate level of symptom load; *** *p* < 0.001.

**Figure 4:**
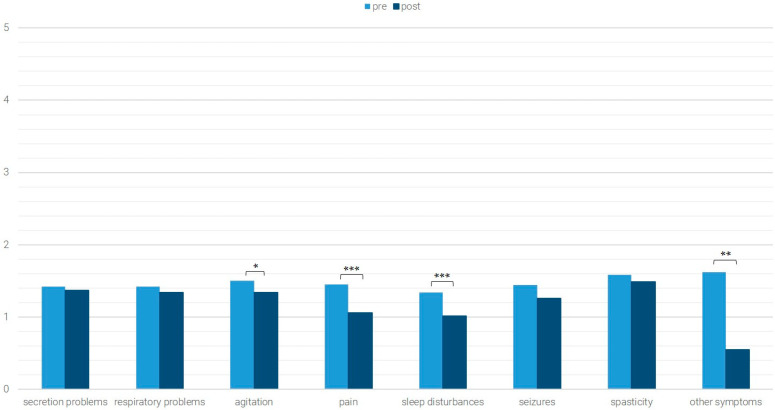
Occurrence of symptoms with an initially low level of symptom load; * *p* < 0.05, ** *p* < 0.01; *** *p* < 0.001.

The average values of the pre- and post-data of the *n* = 574 patients for whom both pre- and post-data were available are shown in Figure 1, Figure 2, Figure 3 and Figure 4. Significant differences (Wilcoxon test with *p* < 0.05) are marked.

### 3.1. Family’s Current Situation

The family’s current situation was descriptively rated more positively at discharge (post-survey) than at admission (pre-survey; Figure 1). Significant improvements were identified for the following items: “The family feels safe in providing care to their child at home” (Z = −4.384, *p* < 0.001, *n* = 569, r = 0.184), “The family knows the child’s symptoms” (Z = −4.649, *p* < 0.001, *n* = 571, r = 0195), “The family is overwhelmed by their child’s care” (Z = −2.693, *p* = 0.007, *n* = 570, r = 0.113), “The family was constantly worried about their child” (Z = −3.898, *p* < 0.001, *n* = 566, r = 0.164), “The family can assess the child’s needs” (Z = −2.178, *p* = 0.029, *n* = 542, r = 0.094), and “In assessing the child, the family trust their gut feeling” (Z = −2.762, *p* = 0.006, *n* = 569, r = 0.116).

Furthermore, the caregiver’s competency also increased significantly between the two measurement points (Z = −4.878, *p* < 0.001, *n* = 571, r = 0.204; Figure 2). The internal consistency for this scale, Caregiver’s Competencies, was acceptable (Cronbach’s alpha = 0.79).

### 3.2. Symptom Burden

At admission, 78% (*n* = 533) of the patients had at least one symptom with at least moderate symptom load. More than half of the patients exhibited pain (60%), agitation (60%), respiratory problems (58%), secretion problems (56%), or spasticity (51%). In addition, 49% had sleep disturbances, and 36% suffered from seizures (Table 2).

Among patients with moderate to initial high symptom burden, the most prevalent symptoms at admission were respiratory problems (59%), spasticity (52%), and secretion problems (49%).

For all symptoms, significant reductions were achieved between admission and discharge (Figure 3): secretion problems (Z = −7.862, *p* < 0.001, *n* = 158, r = 0.625), respiratory problems (Z = −9.147, *p* < 0.001, *n* = 198, r = 0.650), agitation (Z = −9.207, *p* < 0.001, *n* = 167, r = 0.712), pain (Z = −8.946, *p* < 0.001, *n* = 138, r = 0.762), sleep disturbances (Z = −7.517, *p* < 0.001, *n* = 105, r =0.734), seizures (Z = −6.394, *p* < 0.001, *n* = 85, r = 0.694), spasticity (Z = −7.122, *p* < 0.001, *n* = 157, r = 0.568), and other symptoms (Z = −4.956, *p* < 0.001, *n* = 52, r = 0.687).

Patients with initially low symptom burden showed significant improvements in agitation, pain, sleep disturbances, and other symptoms (agitation: Z = −2.023, *p* = 0.043, *n* = 192, r = 0.146; pain: Z = −4.066, *p* < 0.001, *n* = 208, r = 0.282; sleep disturbances: Z = −4.071, *p*< 0.001, *n* = 183, r = 0.301; other symptoms: Z = −2.434, *p* = 0.015, *n* = 11, r = 0.734) (Figure 4).

### 3.3. Global Assessment

**Figure 5:**
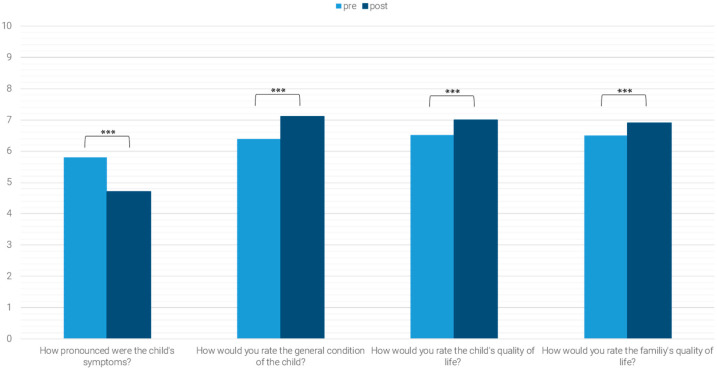
Quality of life; *** *p* < 0.001.

Mean scores for the global assessment items (Part C) also improved significantly between admission and discharge (Figure 5):

“How pronounced were the child’s symptoms?” (Z = −9.750, *p* < 0.001, *n* = 558, r = 0.413), “How do you assess the child’s general condition?” (Z = −6.978, *p* < 0.001, *n* = 555, r = 0.296), “How do you assess the child’s quality of life?” (Z = −4.658, *p* < 0.001, *n* = 553, r = 0.198) and “How do you assess the family’s quality of life?” (Z = −3.948, *p* < 0.001, *n* = 550, r = 0.168) (Figure 5).

## 4. Discussion

Using the assessment tool QUASI, nurses at a specialized 8-bed inpatient PPC unit collected data on three PPC core treatment goals: (i) reduction in symptom burden, (ii) improvement of the quality of life of both the child and their family, and (iii) strengthening caregiver competency. The results demonstrate significant improvements across all assessed dimensions. In conclusion, core treatment goals of PPC can be achieved by specialized inpatient PPC, and finally, high quality care on a PPC unit can be achieved by addressing core treatment goals of PPC.

Although the majority of families receive community-based care or are cared for by Specialized Pediatric Palliative Home Care (SPPHC) Teams, situations may arise that require admission to a PPC unit. The most common reason for admission is high symptom load. At admission, 78% of the patients had at least one symptom with at least moderate symptom load. As reported, a significant reduction in symptom load in all assessed symptoms was achieved during the inpatient stay on the PPC unit. Not least due to the skilled multiprofessional team of the PPC unit that is required to address the wide-ranging symptoms. This is a first indication of reduced symptom burden for patients on the PPC unit.

Fortunately, reduced symptom load is known to contribute to improved quality of life of the affected child and the entire family [10,12,13]. Families with a child with PPC needs are recognized to manage a uniquely stressful set of responsibilities that lead to negative impacts on parental/caregiver quality of life [10,13,14]. Results show that an inpatient stay on the specialized PPC unit leads to an improved quality of life for both the patient and the entire family. Inpatient care may offer families a temporary reprieve, allowing them to rest and regain emotional and physical strength. According to Kittelsen et al. [12], the practical assistance enhances parents’ quality of life as it creates space for the parents for themselves or to engage more fully with siblings. Parents of children with PPC needs strive to create normality in their daily lives [12,15]. The inpatient stay on a specialized PPC unit supports them not only to rest but also to recognize their own resources. Especially the psychosocial team of the PPC unit supports the family in jointly considering what support can be established at home [16]. Furthermore, a recent study by Kittelsen et al. [17], that explored the lived experiences of children living with life-limiting or life-threatening conditions, highlighted that the children’s attention revolved around life rather than their illness. In contrast, parents, as well as professional caregivers, undervalued the child’s opportunities for play and engagement in meaningful activities by focusing on alleviating the clinical symptoms and minimizing psychosocial impact on the child [18]. To achieve core treatment goals in PPC, the children’s desire to engage in life should be acknowledged. Additionally, the dynamic, that the child’s quality of life affects the parent’s quality of life and vice versa [12], should be identified and addressed.

Regarding caregiver competencies, results show a significant increase between admission and discharge from the PPC unit. Parents of children with PPC needs develop expertise in caring for their child’s complex needs; they have vital information about their child’s health and well-being and are experts regarding how their child reacts to illnesses and treatments as well as their child’s physical and emotional needs [19]. Likewise, they often assume the role of a primary care coordinator [13]. However, several studies regarding parents of children with PPC needs illustrate that, especially in the inpatient setting, parents do not feel listened to or heard during the process of decision-making for their child [12,14] and often struggle to obtain the information or advice they need from healthcare professionals [20,21]. Especially, when an inpatient stay on a PPC unit is required, it is important to take the parents seriously, to understand their needs, and to make joint decisions. This will strengthen their caregiver competencies and thereby discharge them empowered. While our findings indicate that the QUASI may be a practical tool for routine PPC documentation, as it covers core dimensions of PPC very effectively, conclusions regarding its broader implementation should be drawn with caution until further psychometric evaluation has been completed. The results provide an initial indication of its potential utility, but further research is needed to confirm its validity and applicability across settings. Furthermore, additional tools (e.g., from nursing or medical complexity research) are required to address the complexity in PPC.

## 5. Limitations

This study has several limitations. First, it was conducted in a single pediatric palliative care unit in Germany, which may limit the generalizability of the findings to other settings, particularly in different healthcare systems or cultural contexts. However, the setting reflects a typical specialized inpatient unit, and the use of routine documentation enhances ecological validity. Future studies should aim to replicate findings in multi-center or international contexts to strengthen external validity.

Second, this study employed the QUASI as a pragmatic assessment tool within routine PPC documentation. Although the tool has not yet undergone psychometric validation as a standalone measure, its core items are derived from the rigorously validated FAC-ETS-OF-PPC and have been chosen to reflect well-established concepts relevant to PPC. Furthermore, our analyses provide initial psychometric evidence: the Caregiver Competencies subscale demonstrated acceptable internal consistency, and a confirmatory factor analysis supported the anticipated one-factor structure with an excellent model fit. These findings strengthen confidence in the applicability of this subscale in clinical practice. Nevertheless, comprehensive psychometric testing of the QUASI as a whole remains necessary, including validation across different domains, care settings, and respondent groups.

Third, the use of exclusively nurse-reported outcomes may introduce a form of self-assessment bias, even though the QUASI items do not explicitly ask for evaluation of staff performance. Nurses, due to their close and continuous contact with patients and families, are well positioned to observe clinical changes and family dynamics. However, parental assessments might differ—particularly regarding perceived symptom relief, emotional burden, or quality of life—based on their subjective experience and expectations. Including parental input in future studies could provide a valuable complementary perspective, help identify potential discrepancies, and further strengthen the validity and depth of outcome assessment in PPC settings. Finally, although the QUASI offers a brief and pragmatic approach to assessing outcomes in pediatric palliative care, it cannot fully capture the complexity and multidimensionality of such care. Complementary tools or qualitative methods may be needed to provide a more comprehensive evaluation in future research.

## 6. Conclusions

In conclusion, core treatment goals of PPC can be achieved by specialized inpatient PPC, and finally, high-quality care on a PPC unit can be achieved by addressing core treatment goals of PPC. Additionally, QUASI is a highly suitable tool for the assessment of core dimensions of PPC in an inpatient setting. To address the complexity of PPC, additional tools are required, as well as further studies in other institutions/settings, which are of great interest in order to continuously improve care in PPC.

## Figures and Tables

**Table 1:** Patients’ underlying diseases ^1^.

Underlying Diseases ^1^	*N* (%)
Neuromuscular	289 (42.8)
Premature or Neonatal	136 (19.9)
Other Congenital or Genetic Defect	120 (17.6)
Metabolic	91 (13.3)
Malignancy	25 (3.7)
Cardiovascular	9 (1.3)
Gastrointestinal	4 (0.6)
Heamatologic or Immunologic	1 (0.1)

^1^ According to the system of pediatric complex chronic conditions (version 3) by Feudtner [11].

**Table 2:** Overview of all QUASI items.

Item			Pre				Post			
			*N*	Median	Mean	SD	*N*	Median	Mean	SD
Family’s current situation	The family feels safe in providing care to the child at home		672	4	3.61	1.1	580	4	3.8	1.03
	The family knows the child’s symptoms		673	4	3.82	0.95	580	4	4.0	0.89
	The family is overwhelmed by their child’s care		673	2	2.06	1.35	580	2	1.92	1.27
	The family was constantly worried about their child		667	3	2.26	1.31	577	2	2.35	1.32
	The family can assess the child’s needs		670	4	3.71	0.92	555	4	3.8	0.96
	In assessing the child, the family trusts their gut feeling		670	4	3.62	0.94	581	4	3.74	0.93
	The family is convinced that they make the right decisions for the child		669	4	3.67	0.88	575	4	3.73	0.88
	Caregiver’s Competency		673		3.19	0.96	581		3.39	0.925
		If necessary, the family is able to independently take measures to alleviate their child’s symptoms	673	3	3.33	1.02	577	4	3.58	1.0
		The family is prepared for the child’s crisis	670	3	3.03	1.09	578	3	3.26	1.02
		The family has a clear idea of what should be done for their child in a medical emergency	670	3	3.29	1.17	578	3	3.42	1.11
symptoms	Secretion problems		382	2	2.47	1.24	318	2	2.29	1.01
	Respiratory problems		397	3	2.82	1.33	330	2	2.47	1.24
	Agitation		414	2	2.46	1.25	344	2	2.19	1.09
	Pain		414	2	2.21	1.13	290	2	1.99	1.0
	Sleep disturbances		336	2	2.16	1.19	267	2	2.09	1.08
	Seizures		234	2	2.27	1.15	220	2	2.12	0.963
	Spasticity		349	2	2.69	1.29	315	2	2.46	1.21
	Other symptoms		133	3	3.24	1.07	81	3	2.95	1.19
Global assessment	How pronounced were the child’s symptoms?		668	6	5.66	2.28	570	5	4.75	2.01
	How would you rate the general condition of the child?		667	7	6.40	2.00	568	7	7.09	1.76
	How would you rate the child’s quality of life?		664	7	6.50	2.05	569	7	6.96	1.86
	How would you rate the family’s quality of life?		659	7	6.43	1.99	565	7	6.86	1.79

## Data Availability

The datasets used and/or analyzed within the framework of this study are available from the corresponding author on reasonable request.
